# Viscoelastic Response of Elastohydrodynamically Lubricated Compliant Contacts below Glass-Transition Temperature

**DOI:** 10.3390/polym15112528

**Published:** 2023-05-30

**Authors:** Jiri Krupka, Krystof Dockal, Tomas Sedlacek, David Rebenda, Ivan Krupka, Martin Hartl

**Affiliations:** 1 Faculty of Mechanical Engineering, Brno University of Technology, Technicka 2896/2, 616 69 Brno, Czech Republic or rebenda@utb.cz (D.R.); krupka@fme.vutbr.cz (I.K.); hartl@fme.vutbr.cz (M.H.); 2 HVM Plasma spol. s.r.o., Na Hutmance 2, 158 00 Praha 5, Czech Republic; krystof.dockal@hvm.cz; 3 Centre of Polymer Systems, Tomas Bata University in Zlin, Trida Tomase Bati 5678, 760 01 Zlin, Czech Republic; sedlacek@utb.cz; 4 Footwear Research Centre, University Institute, Tomas Bata University in Zlin, Nad Ovcirnou IV 3685, 760 01 Zlin, Czech Republic

**Keywords:** compliant contact, elastohydrodynamic lubrication, transition region, fluid-film thickness, optical chromatic interferometry, viscoelastic behavior

## Abstract

The widespread use of polymers in the high-performance engineering applications brings challenges in the field of liquid lubrication in order to separate the rubbing surfaces by the coherent fluid-film thickness relative to not only the inelastic material response of the polymers. The determination of the mechanical properties by the nanoindentation and the dynamic mechanical analysis represents the key methodology to identify the viscoelastic behavior with respect to the intense frequency and temperature dependance exhibited by polymers. The fluid-film thickness was examined by the optical chromatic interferometry on the rotational tribometer in the ball-on-disc configuration. Based on the experiments performed, first, the complex modulus and the damping factor for the PMMA polymer describing the frequency and temperature dependence were obtained. Afterwards, the central as well as minimum fluid-film thickness were investigated. The results revealed the operation of the compliant circular contact in the transition region very close to the boundary between the Piezoviscous-elastic and Isoviscous-elastic modes of the elastohydrodynamic lubrication regime, and a significant deviation of the fluid-film thickness from the prediction models for both modes in dependence on the inlet temperature.

## 1. Introduction

After the WW2, the first phase of the widespread use of polymers to many areas of industry was recorded [1,2]. Originally, the polymer machine elements, such as gears, were mainly operated under dry conditions or lubricated by greases and used to transfer the motion rather than to transmit torque. Conversely, the current research in the field of tribology of polymer materials is focused on the operation of machine elements under liquid lubrication conditions, especially, in the elastohydrodynamic lubrication (EHL) regime [3,4,5,6,7,8], where the rubbing surfaces are fully separated by a coherent film thickness of the lubricant.

Nowadays, the polymers in connection with the EHL regime make available the implementation into the high-performance applications where dynamics, tribology and extreme operating conditions interact. Transmissions, differentials and mechanisms with polymer gears are examples of the use in automotive, aviation, space industries, etc. One of the constrains of polymers is a significant dependence of mechanical properties on the temperature, which leads to the manifestation of viscoelastic behavior. In terms of tribology, the question arises, how the viscoelastic response of the polymer affects the formation of film thickness in compliant contacts operated in the EHL regime under operating conditions far from the glass-transition temperature of the polymer selected.

The current paper is concerned with the basic oriented research. The main aim of this paper is to investigate the formation of fluid-film thickness in the compliant circular contact operating in the EHL regime with respect to the temperature and time (or frequency), which characterize the viscoelastic response of the polymer. For validation, the amorphous transparent polymer Polymethyl-methacrylate (PMMA) and the reference synthetic lubricant 5P4E were employed. The results are presented in part A and part B where the former deals with the analysis of the material properties of PMMA by nanoindentation (nano-DMA) and the dynamic mechanical analysis (DMA). The latter is concerned with the analysis of the fluid-film thickness in compliant circular contact using the method of optical chromatic interferometry. The subject of this paper is further described in the Supplementary Information (SI)—A.

## 2. Materials and Methods

### 2.1. Selection of Materials

In part A, a plate-shaped PMMA polymer specimens of 10 (w) × 10 (l) × 0.5 (t) mm were used for determination of mechanical properties based on the nano-DMA. Moreover, the cylinder-shaped PMMA specimens of 3 mm in diameter and 8 mm in length for DMA were used. In Part B, a compliant contact was simulated between the flat PMMA polymer disc with a diameter of 120 mm, and the 100Cr6 bearing steel ball of 25.4 mm in external diameter. The purchased XT PMMA sheets (NUDEC, S. A (ES)) were manufactured by an extrusion process. The amorphous PMMA disposes of excellent light transmittance, over 90%, necessary for implementation of optical methods.

To achieve a sufficient interference of light, and to avoid the presence of parasitic light, the PMMA disc was coated with semi-reflective chromium and antireflective layers vapor-deposited on the bottom side (in contact with ball) and on the top side of disc, respectively. The mechanical properties of PMMA [9,10] are close in values to the engineering [11] and high-performance [12] polymers (PA66, POM, PEEK) frequently used to produce machine elements. Selected properties of specimens, such as RMS roughness (R_q_) or E, are stated in Table 1.

### 2.2. Selection of Lubricants

In part B, the compliant contact was lubricated with Santovac^®^ 5—Polyphenyl Ether (5P4E), a synthetic diffusion pump oil produced by SANTOLUBES LLC company (Spartanburg, SC, USA). The selected lubricant belongs to the API (American Petroleum Institute, Washington, DC, USA) Group V according to the base oil categories including synthetic lubricants such as polyglycols, silicones and esters. One of the main characteristics of 5P4E lubricant is its extreme viscosity, approximately η_0_ ≈ 3 Pa s at 24 °C.

Hence, it is usually used in aviation and aerospace applications for extreme environmental and operating conditions. In addition, the 5P4E is frequently used as a reference lubricant in the field of EHL [7,13]. The rheological properties of 5P4E, such as the dynamic viscosity at atmospheric pressure (η_0_) and pressure–viscosity coefficient (α) at 40 and 100 °C, are given in Table 2.

### 2.3. Experimental Apparatus

Four commercial and one non-commercial experimental apparatuses were employed to determine the properties of PMMA, to evaluate the surface topography of selected specimens and the film thickness of the lubricant in compliant contact. In part A, Hysitron TI Premier Nanoindenter (Bruker) was used to analyze mechanical properties. For the chemical analysis, we employed the Raman spectroscopy using the Renishaw inVia Reflex Raman spectroscope and differential scanning colorimetry (DSC) by TA Instruments. DMA describing the viscoelastic properties of studied PMMA specimen was carried out in laboratories of the Centre of Polymer Systems (CPS) using a dynamic mechanical analyzer DMA1 (Mettler Toledo, Switzerland) equipped with clamps for compression testing mode.

In part B, the 3D optical profilometer Contour GT-X by Bruker was utilized for the surface texture analysis. For the analysis of the fluid-film thickness in compliant circular contact, the rotary optical tribometer in ball-on-disc configuration, developed by the tribology group of Brno University of Technology (BUT) in the Czech Republic, was employed. This experimental apparatus has recently been described by the authors in greater detail in [14,15].

### 2.4. Experimental Conditions and Methods

In part A, the nano-DMA experiments of PMMA were performed to determine the E′ and E″ moduli and the tan δ, see Equations (SA8) and (SA9). The experimental conditions for part A are stated in Table 3. The nanoindentation technique nanoDMA III on the Bruker Hysitron Premier Ti nano-indenter was used to measure the dynamic mechanical properties of PMMA. Two types of experiments were conducted with a maximum load (by normal force) of 13 mN at a temperature interval from 15 to 80 °C for the six discrete temperatures.

The first experiment consisted of a frequency sweep from 1 Hz to 250 Hz, and the second involved CMX to obtain a modulus at different indentation depths. The load rate was 1.3 mN/s, while an ideal Berkovich indenter with a tip radius of 150 nm was used for all tests. During each experiment, the sample was kept in the xSol temperature stage. Secondly, PMMA cylinder specimens were employed for the DMA frequency sweep analysis in the frequency range between 10^−3^ and 10^2^ with five measuring points per decade. Based on the preliminary results of the amplitude dependent viscoelastic characterization, the linear viscoelastic region (deformation amplitude of 3 μm) was defined and applied for further experiments at specified temperatures.

Based on frequency sweep experiments, the TTSP principle was applied to obtain an MC that extends the frequency range beyond the measured frequency (f). Then, the MC was regressed according to the WLF equation (Equation (SA11)), and model parameters were derived. Besides this, the T_g_ region was measured by DSC at heating rate of 10 °C/min. Raman spectra of PMMA were collected before and after the tribological experiments as well. To obtain these spectra, the laser 785 nm was used.

In part B, prior to the film thickness experiments, the 3D optical profilometer was employed for the surface structure analysis. The PMMA disc has an optically smooth surface, see Table 1; however, the steel ball was repeatably polished to decrease the R_q_ and to get rid of parasitic optical reflectivity. After the preparation steps, the optical chromatic interferometry was implemented for evaluation of the h_c_ and h_m_ in the compliant contact. A detailed description of this method could be found in [16]. The fluid-film thickness was measured, calibrated, and evaluated in software Achilles (ver. 4.0.117, Radek Poliscuk, Brno University of Technology, Brno, Czech Republic).

The h_c_ and h_m_ formed in the compliant contact between the PMMA disc and the steel ball were evaluated under a single constant load, W = 35 N. Hence, the contact pair formed a circular contact with respect to the Hertzian theory corresponding to the ellipticity, *k* = 1. The temperature was controlled by thermocouples in the oil reservoir and thermocouples placed in the vicinity of inlet of the lubricant to the contact, which corresponds to the oil temperature and the inlet temperature (T), respectively. Four discrete inlet temperatures T from 24 to 80 °C below the widely used T_g_ interval (105–110 °C) [17] of PMMA were selected.

The experiments were carried out on the rotary optical tribometer under the pure rolling conditions where U_1_ = U_2_ (1 − disc, 2 − ball) corresponding to the sliding-rolling ratio SRR = 0. Therefore, the entrainment speed U_E_ is equal to the surface speed of the PMMA disc U_1_ as well as of the steel ball U_2_. Based on this, the frequency of loading f_L_ could be expressed by the ratio of U_E_ and the Hertzian contact radius a_H_, see Equation (1), which corresponds to the state of loading and unloading of the contact during the one cycle.

(1) $$f_{L}=\frac{U_{E}}{a_{H}}$$

Utilizing the 5P4E lubricant, the influence of U_E_ and T on h_c_ and h_m_ was determined under the condition of isothermal full-film separation of contact surfaces. The film thickness was measured in the ascending order relative to the U_E_. Four discrete values of h_c_ (100, 150, 200 and 250 nm) were selected for which the profiles of film thickness in transverse (x) and longitudinal (y) direction to U_E_ were assessed.

To detect a possible transition of h_m_ [18], the minimum film thickness was evaluated from interferograms at the exit of contact h_mr_ as well as at the side lobes h_ms_ of horseshoe. After collecting the experimental data, h_c_ and h_m_ were compared with the soft [18,19,20] and hard [5] EHL prediction models including Equations (SA4)–(SA7), and differences were discussed. Moreover, the operation region of the contact was identified relative to the EHL modes via a hydrodynamic map [21,22] by implementation of Equations (SA1)–(SA3).

The material response of the PMMA polymer was determined from the interferograms of initially circular contact at the individual T. Thus, the variations in R_c_, ellipticity *k* and contact area *A* for static (U_E_ = 0) as well as running (U_E_ ≠ 0) contacts were evaluated. For the comparison, the maximal Hertzian contact pressures, p_H_ and a_H_, were calculated according to Equations (2) and (3) as 56 MPa and 443 µm at 24 °C, respectively.

(2) $$p_{H}=\frac{3W}{2{\pi \;a}_{H}{}^{2}}$$

(3) $$a_{H}=\sqrt[{3}]{\frac{3\mathrm{WR}}{2E_{R}}}$$

(4) $$\frac{2}{E_{R}}=\frac{\left(1-\upsilon_{1}^{2}\right)}{E_{1}}+\frac{\left(1-\upsilon_{2}^{2}\right)}{E_{2}}$$

Subsequently, the reduced elastic modulus ($E_{R}$) and the elastic modulus of PMMA (E_1_) were reversibly calculated from Equations (3) and (4) for individual T after the a_H_ was substituted by the experimentally obtained R_c_. Nevertheless, these values were determined for the static contact (U_E_ = 0) assuming a pure elastic response of the PMMA disc, which did not manifest the effects of viscoelastic behavior under dynamic cyclic loading. The experimental conditions for part B are summarized in Table 4.

## 3. Results

### 3.1. Part A. Analysis of Material Properties of PMMA

From the part A, the frequency and temperature dependence of PMMA was determined employing the nanoindentation and the dynamic mechanical analysis considering the experimental conditions, see Table 3. To characterize the response of the material, five parameters—elastic E, storage E′, loss E″ and complex E^*^ (see Equation (SA8)) moduli and damping factor tan (δ) (see Equation (SA9))—were measured below T_g_, which reflects the experimental conditions of tribological experiments in part B, see Table 4. The glass-transition for the PMMA specimens was determined by DSC corresponding to the interval $T_{g\;}\in \;❬104,\;115❭$ °C. This procedure was repeated before and after tribological experiments in part B together with acquisition of the Raman spectra to detect the possible changes in the PMMA structure. However, none of the measurements showed any significant variations during the tribological experiments.

**Figure 1:**
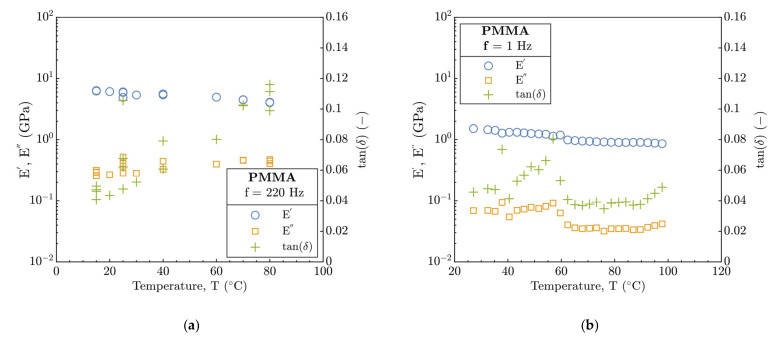
Temperature dependence of storage E′ and loss E″ moduli, and damping factor tan (δ) for pure PMMA at constant reference frequency: (**a**) nano-DMA, (**b**) DMA.

The temperature dependence of E′ and E″ moduli and tan (δ) was determined at the constant reference frequency of 220 Hz and 1 Hz for nano-DMA and DMA, respectively, see Figure 1a,b. For the former and latter, frequency sweep and frequency as well as temperature sweep tests were performed. As expected, the values obtained from DMA are significantly lower compared to those from nano-DMA due to a different scale of the experiment. The E′ exhibited in both analyses a gradual linear decrease in the whole measured temperature range. The E″ obtained by nano-DMA demonstrated only a slight but gradual increase with temperature where for the DMA data, such increase begins only at 80 °C. The tan (δ) develops in similar manner relative to the E″ with minimal value of approx. 0.04.

In Figure 1b, using the DMA analysis, the E″ and tan (δ) data exhibit a significant fluctuation between 40–60 °C; however, E′ qualitatively corresponds to the nano-DMA data. Quantitatively, E′ and E″ moduli from nano-DMA overrated the data from the DMA temperature sweep tests several times. Conversely to E″ and E′, the data of tan (δ) were in accordance with the results in Figure 1a.

**Figure 2:**
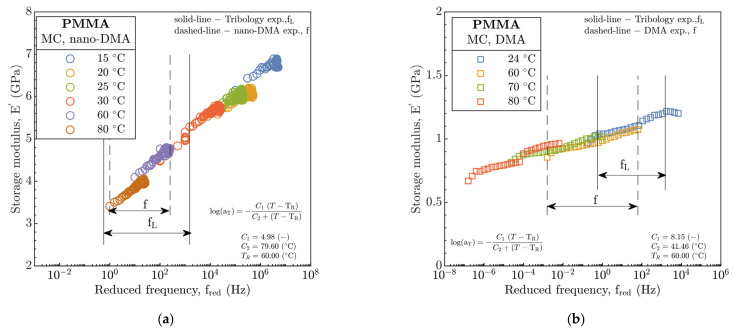
Master-curve of storage modulus E′ at reference temperature T_R_ = 60 °C. (**a**) nano-DMA, (**b**) DMA.

The nano-DMA and DMA frequency sweep experiments were also performed in the frequency interval of f∈ ❬1, 250❭ Hz and $f\in \;❬10^{-3},\;10^{2}❭$ Hz for several discrete temperatures. This provided necessary input data to compose the MC by implementation of the TTSP. The MC were derived for E′, see Figure 2a,b, at ${\;T}_{R\;}=60\;{}^{\circ}C$ on account of the supplementation of this parameter into the tribological prediction models of h_c_ and h_m_ in part B.

After implementation of TTSP, the reduced frequency (f_red_) extended the operating frequency range from $10^{0}$ to $10^{7}$ for the nano-DMA and from $10^{-7}$ to $10^{4}$ for the DMA analysis. However, the significant difference of nano-DMA and DMA data relative frequency (or temperature) is quite evident. The former corresponds to the increase in E′ from 3 to 7 GPa, for the latter, E′ was noticeable lower, ranging only between 0.5 and 1.5 GPa with increase in f_red_.

The superposition of the data highlighted a difference between the operating frequency of nano-DMA as well as DMA experiments in part A (see Table 3) and tribological experiments in part B (see Table 4, evaluated according to Equation (1)). Hence, this justified using of the TTSP together with derivation of a_T_ that were regressed according to the WLF equation, see (Equation (SA11)). Then, the constants C_1_ and C_2_ were obtained, see Figure 2a,b.

### 3.2. Part B. Analysis of Fluid-Film Thickness in Compliant Contact

**Figure 3:**
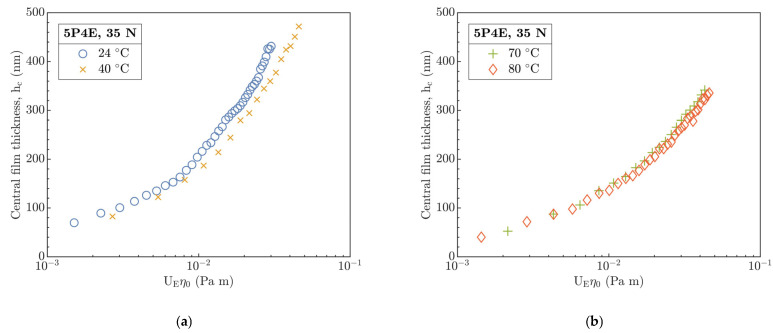
Central film thickness h_c_ versus product of U_E_η_0_ at different inlet temperatures. Temperatures (**a**) 24 and 40 °C, (**b**) 70 and 80 °C.

From the part B, data of the h_c_ and h_m_ were evaluated for experimental conditions, see Table 4. At first, interferograms were centered in x-y coordinates to determine the center of the contact at the intersection of horizontal and vertical paths according to the Newton’s fringes. Then, h_c_ was evaluated relative to the product of entrainment speed U_E_ and dynamic viscosity η_0_, as Figure 3a (24 and 40 °C) and Figure 3b (70 and 80 °C) demonstrate.

From Figure 3a, it is evident that h_c_ qualitatively corresponds well, but it quantitatively differs with increase in U_E_η_0_. On the other hand, in Figure 3b, h_c_ differs only slightly in the values, and the development of h_c_ at 70 and 80 °C in dependence on U_E_η_0_ is practically identical. In both figures, the product of U_E_η_0_ spans a similar range from $10^{-3}$ to $10^{-1}$ Pa·m in the entire temperature interval corresponding to h_c_ < 500 nm. Nevertheless, the absolute difference of h_c_ develops in a dissimilar manner between 24 and 40 °C as well as between 70 and 80 °C relative to the product of U_E_η_0_.

**Figure 4:**
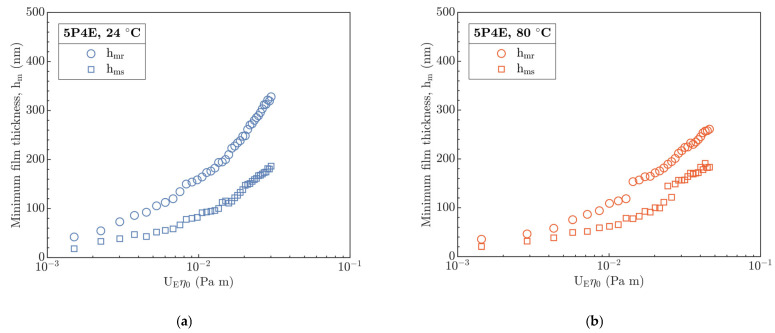
Minimum film thickness at contact exit h_mr_ and on side lobes h_ms_ versus product of U_E_η_0_ at different inlet temperatures. (**a**) 24 °C, (**b**) 80 °C.

The minimum film thickness was identified at the side lobes of the contact h_ms_ without no signs of transition of the minimum to the exit h_mr_ in all experiments, see Figure 4a,b. The difference between h_mr_ and h_ms_ gradually decreases with the increasing temperature. The increase in temperature (T ≥ 40 °C) at lower values of U_E_η_0_ (up to 10^−2^) demonstrated a tendency of h_mr_ and h_ms_ to unify into a single curve; however, at higher values of U_E_η_0_, this led to the increase in the variation between h_mr_ and h_ms_.

**Figure 5:**
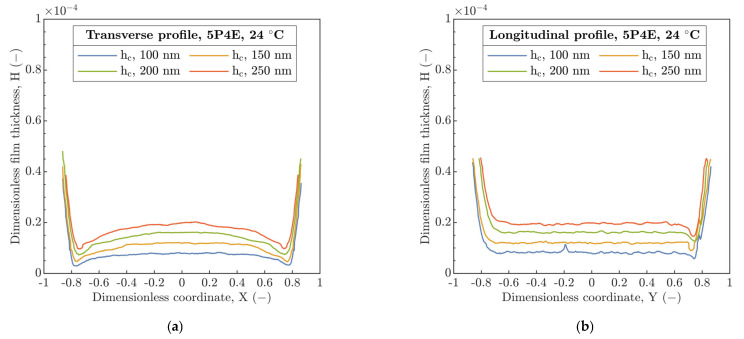
Film thickness profiles along the contact center line at 24 °C. (**a**) Transverse profile, (**b**) longitudinal profile.

**Figure 6:**
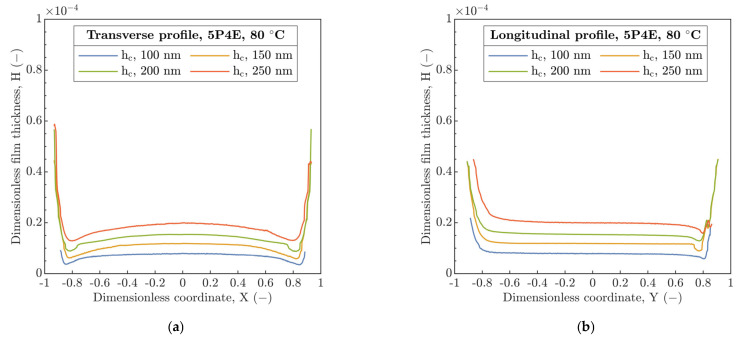
Film thickness profiles along the contact center line at 80 °C. (**a**) Transverse, (**b**) longitudinal.

In the next step, the film thickness profiles were investigated in the perpendicular and parallel direction relative to U_E_ along the center line of the contact for four discrete h_c_ (100, 150, 200 and 250 nm), see Figure 5 and Figure 6 showing dimensionless coordinates X and Y versus film thickness H. At 24 °C, the coherent film thickness is formed and the horseshoe shape with constriction at the side lobes is evident from the transverse profile in Figure 5a. An increase in U_E_ led to a more pronounced horseshoe film shape, so the difference between h_c_ and h_ms_ is more significant. In the parallel direction to U_E_, the film thickness profile along the center line was almost constant except for the contact inlet (on the left) and for the exit from the contact (on the right) where, for the latter, significant variations in the wedge shape were observed, see Figure 5b.

Contrary to thickness profiles obtained at 24 °C, an increase in inlet temperature to 80 °C, see Figure 6a results in a reduction in the side lobes of horseshoe. Moreover, the gradual change in the film thickness in the transverse profile was observed in the direction from the side lobes to the central region along the center line, which corresponds to the increase in the ratio h_ms_/h_c_.

The film thickness profiles parallel to U_E_, see Figure 6b, demonstrate a similar behavior such as at 24 °C, without inclination of film thickness profile with increasing U_E_. In addition, the increase in T probably initializes the polymer constitutive variation in material stiffness connected with a change in the pressure distribution and thus the asymmetrical change in the radius of running contact R_c_ in the transverse and longitudinal profiles. However, for the former, this deviation is more noticeable. In the longitudinal profiles at the contact inlet, the secondary local minimum of film thickness was not recorded.

Further results represent the evaluation of interferograms of static contacts (U_E_ = 0) at different temperatures. The results summarized in Table 5 showed a linear increase in R_c_ from 24 °C to 80 °C where the absolute difference represents the increase in R_c_, approximately by 17%. Moreover, the difference of R_cx_ and R_cy_ in the transverse and longitudinal directions, respectively, was almost negligible.

Apart from R_c_, the area of contact *A* demonstrated a significant enlargement (about 30%) when the inlet temperature increased from 24 °C to 80 °C. R_cx_ and R_cy_ were averaged and then substituted into Equations (3) and (4), from which the E_R_ and E_1_ were calculated, see Table 6.

**Figure 7:**
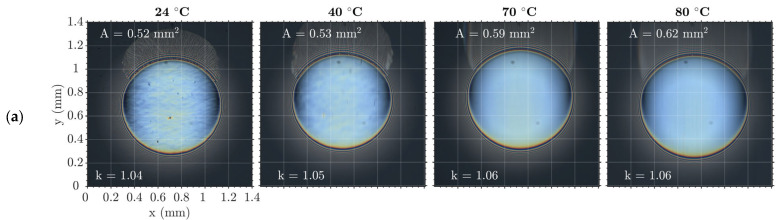

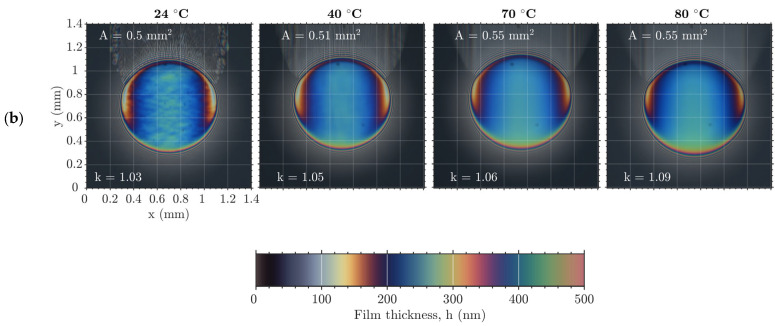
Interferograms of running contact (U_E_ ≠ 0) at different inlet temperatures for h_c_: (**a**) 100 nm, (**b**) 250 nm.

On the other hand, the running contact (U_E_ ≠ 0) was always dimensionally smaller exhibiting a proportional difference of R_c_ between the transverse and longitudinal directions in the entire temperature interval relative to the static contact (U_E_ = 0) see Figure 7. This results in the ellipticity variation in the interval $k\;\in \;\left(1.03,\;1.10\right)$ where higher values of *k* correspond with enhancement in inlet temperature. Only a slight transition from the circular (*k* = 1) to the wide elliptical contact (*k* > 1) was recorded. Despite the increase in ellipticity, the contact area of the running contact *A* was reduced in size by up to 12% in dependence on U_E_ for individual inlet temperatures. Contrary to this, the effect of temperature is more significant than U_E_ throughout the selected h_c_ (100, 150, 200 and 250 nm), which leads to the enlargement of contact area *A* of running contact in the interval from 0.5 to 20% relative to the contact area at 24 °C. However, this difference gradually decreases with increasing h_c_, see Figure 7a,b.

## 4. Discussion

One of the key attributes for operation of compliant contacts in demanding applications is sufficient lubrication of the rubbing surfaces. However, the interaction between viscous liquids and viscoelastic solids represents a complex problem not only relative to the state of the art in the soft EHL. Furthermore, the complexity of this interaction is even more enhanced by significantly different material properties of the rubbing surfaces forming a contact pair.

**Figure 8:**
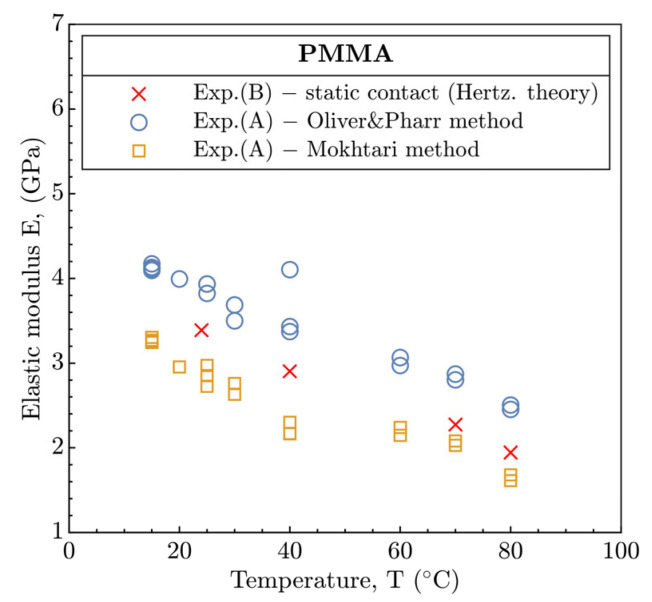
Elastic modulus E according to nanoindentation experiments (Section 3.1. part A) and tribological experiments (Section 3.2. part B).

Generally, in the EHL regime, the constitutive material behavior is described according to the Hertzian theory by the elastic modulus E and the Poisson’s ratio v of the individual solids. In relation to the tribology, the Hertzian theory corresponds to the elastic response of the material and is usually compared with static contacts (U_E_ = 0) to evaluate the contact shape (Equation (3)) and magnitude (Equation (2)) and distribution of the contact pressure. To determine the elastic modulus of PMMA, the nanoindentation experiments in part A were performed and the obtained data were evaluated according to the Oliver and Pharr (O&P) [23] as well as the approach recently described by Mokhtari [24], see Figure 8. Since the data obtained from the nanoindentation may generally be overestimated, the elastic modulus of PMMA disc E_1_ in part B was reversible calculated (see Equations (SA3) and (SA4)) from the radius of static contacts R_c_ (see Table 5) at the individual inlet temperatures T, see Table 6. However, the E_1_ could be influenced by creep of PMMA after the contact is loaded, causing a gradual increase in the contact in size.

Moreover, the evaluation methodology by Mokhtari was included. This demonstrated the development of E in the similar manner as for O&P, but the E was quantitively underestimated considering the results from the tribological experiments in part B, see Figure 8. In comparison, at 24 °C, the E based on the Mokhtari method in part A and part B is in accordance with [10,25], nonetheless, the results of the O&P method in part A differ significantly.

**Figure 9:**
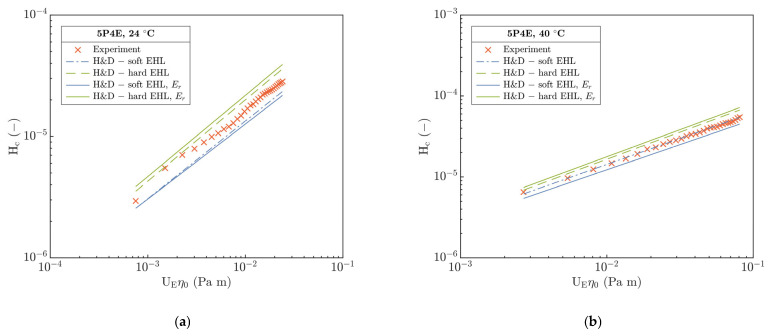
Comparison of experimental dimensionless central film thickness H_c_ versus soft and hard H&D models at different temperatures. (**a**) 24 °C, (**b**) 40 °C; dashed line—H&D, solid line—H&D_m_.

**Figure 10:**
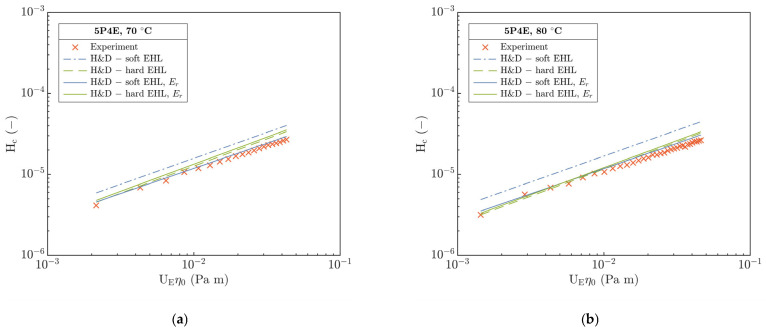
Comparison of experimental dimensionless central film thickness H_c_ versus soft and hard H&D models at different temperatures. (**a**) 70 °C, (**b**) 80 °C; dashed-line—H&D, solid-line—H&D_m_.

Based on the results in part B, the central (see Figure 3) and minimum (see Figure 4) film thickness in the contact of PMMA disc and steel ball were evaluated in dependence on the entrainment speed U_E_ and the inlet temperature T. A comparison of experimental data of central film thickness with the EHL prediction models by Hamrock and Dowson (H&D) for hard [5] (P-E, see Equation (SA4)) and soft [19] (I-E, see Equation (SA6)) EHL is demonstrated in Figure 9 and Figure 10 where the E_1_ from Table 6 was integrated. Both figures show a dependence of H_c_ on the product of U_E_η_0_.

At 24 °C, Figure 9a clearly shows a non-predisposition of experimental data to the soft as well as hard H&D, though H_c_ qualitatively corresponds better with the soft H&D with a difference of 19%. The measured H_c_ relative to the soft model is overestimated; however, for the hard one, it is underestimated. This may result from a significant viscosity effect of used 5P4E lubricant (see Table 2) and only from a slight influence of temperature on the change in the mechanical properties of the polymer at 24 °C.

After the temperature increases to 40 °C, H_c_ correlates best with soft H&D by difference of ca. 2%. Moreover, the deviation of measured H_c_ from hard H&D slightly decreased as well as the deviation of predicted H_c_ between the soft and hard H&D. This could be a consequence of several interconnected effects—a decrease in η and the E with temperature and as a result of decrease in the contact pressure. One of other explanations for the difference between the experimental data and the soft H&D model especially at 24 °C may be a pressure–viscosity effect of the lubricant which correlates with the increase in the film thickness. However, for soft H&D, it is assumed that the effect of α is insufficient due to the expected low contact pressures in the I-E mode of EHL. Hence, $\bar{G}$ (E_R_ α) parameter is omitted from the soft EHL models, see Equation (SA6).

Interestingly, at temperatures of 70 and 80 °C in Figure 10a,b, a good qualitative and quantitative agreement of experimental H_c_ with hard H&D model was observed. However, relative to soft H&D, the measured H_c_ is several times lower than the predicted H_c_, which is in contradiction to the expected behavior with respect to the enhanced compliance of the contact as the temperature increased. Moreover, the η decreased about two orders of magnitude relative to its value at 24 °C.

With the increase in temperature from 70 to 80 °C, the difference in H_c_ between the hard and soft H&D was even more emphasized. A difference between the experimental H_c_ and the hard H&D is only 16% on the average compared to soft H&D, where it exceeds 60%. This points out a discrepancy that, after the temperature increases, the contact of a steel ball and PMMA disc should become more compliant as a consequence of a decrease in E, which corresponds well with the operation in the I-E mode of EHL. However, a decrease in E and thus an increase in contact compliance should be reflected by an increase in H_c_. The origin of this discrepancy seems to be not clear yet.

**Figure 11:**
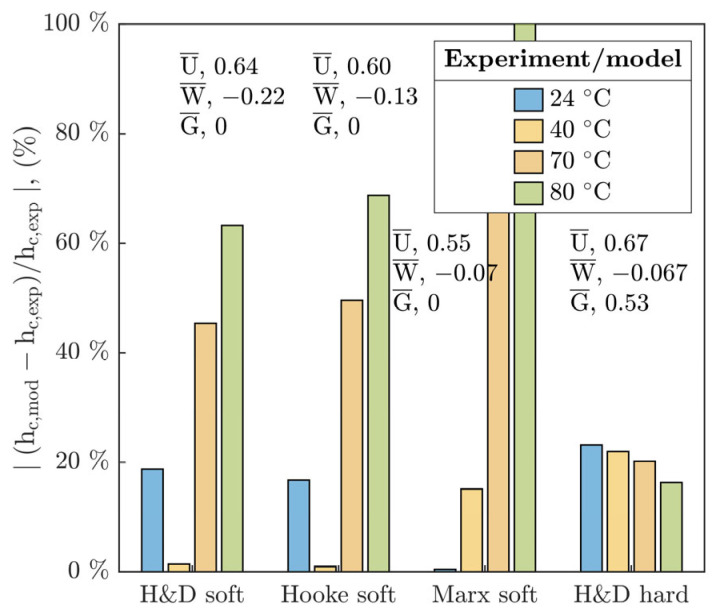
Comparison of central film thickness obtained from the experiments h_c,exp_ and EHL models h_c,mod_. Soft EHL: H&D [19], Hooke [18] and Marx [20]. Hard EHL: H&D hard [5].

Besides the H&D models [5,19], the experimental data of h_c_ and h_m_ were compared with the Hooke et al. [18] and Marx et al. [20] soft EHL models. A discrete interpretation of the deviations of experiments relative to the prediction models is summarized in Figure 11. Here, the main deviation is manifested at 70 and 80 °C; however, it is considerably higher than for the hard H&D, especially for the soft Marx’s and Hooke’s models where it exceeds 50%. Opposite to this, at the temperature of 24 °C, the Marx’s model corresponds well with only a slight deviation up to 3% of the average.

Surprisingly, at 40 °C, the H&D and Hooke’s soft models best match with the experimental H_c_. Generally, the best agreement is demonstrated by the hard H&D with deviation of ca. 15% in the entire temperature interval. In addition, a discrete evaluation of the deviations of the minimum film thickness at the exit of the contact h_mr,exp_ relative to h_mr,mod_ (for H&D see Equations (SA5) and (SA7)) demonstrated a higher deviation than for h_c,exp_ except for the hard H&D.

Comparing the results with EHL models, it is clear that, at high temperatures, the measured thicknesses are different from those predicted. There are several possibilities of how to explain this discrepancy. One of them may be the difference between the material properties and the assumptions of the models including the material parameters such as the E and the v, both defined for Hookean material. However, PMMA is a viscoelastic material whose properties are strongly dependent on time—loading rate, magnitude of strain under loading, and temperature. Nevertheless, these properties are not considered in the EHL models where their influence could be expected especially for the compliant contacts.

**Figure 12:**
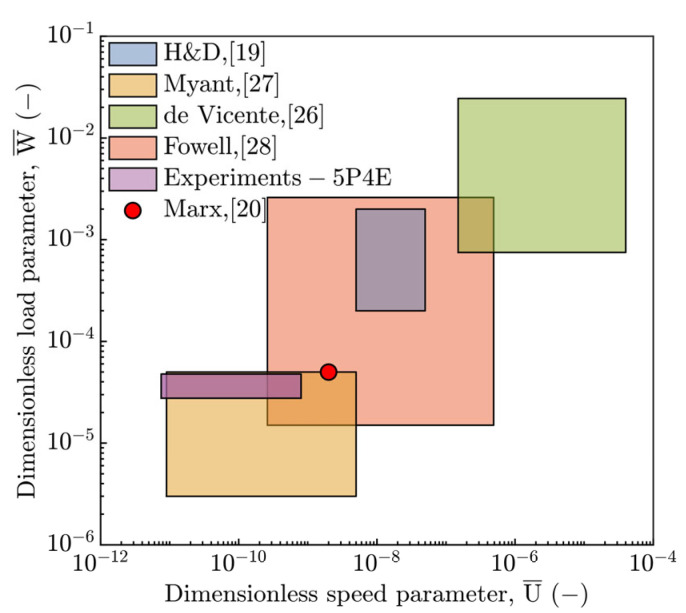
Comparison of the experimental conditions via $\bar{U}$ and $\bar{W}$ parameters in relation to the research in the field of soft EHL.

Observed deviations of h_c_ and h_m_ from the soft EHL models put into the contrast the operating conditions for which the individual prediction formulae were numerically derived or experimentally regressed and for which they are potentially valid. With respect to this, the operating region of experiments was compared with other works [19,20,26] in the field of soft EHL according to the speed $\bar{U}$ and load $\bar{W}$ parameters, see Figure 12. This figure shows a possible source of disagreement of fluid-film thickness between the current study and the H&D model [19], especially for temperature T ≥ 40 °C, where the increase in T corresponds with the increase in $\bar{W}$ parameter. It is evident that the operation region does not match with soft H&D model.

The present study (purple rectangle) almost fully corresponds to the study by Myant et al. [27] according to the speed $\bar{U}$ as well as load $\bar{W}$ parameter. On the other hand, the current study is consistent with the results published by Fowell et al. [28] with respect to the $\bar{W}$. However, relative to the $\bar{U}$, it partially differs. The experimental conditions by de Vicente et al. [26] are shifted to higher values of $\bar{U}$ as well as $\bar{W}$ and thus they considerably differ, see Figure 12.

Nevertheless, the research of the other authors corresponds to a considerably different material configuration of contact pair, except for the study by Marx et al. [20], where the used elastomers and polymers, such as PDMS, exhibit a significant compliance. This reveals a difference from the current study as for mechanical properties of PMMA, see Table 1. Moreover, in these studies, the contact was often operated only up to 40 °C, where the E is not radically affected by temperature.

**Figure 13:**
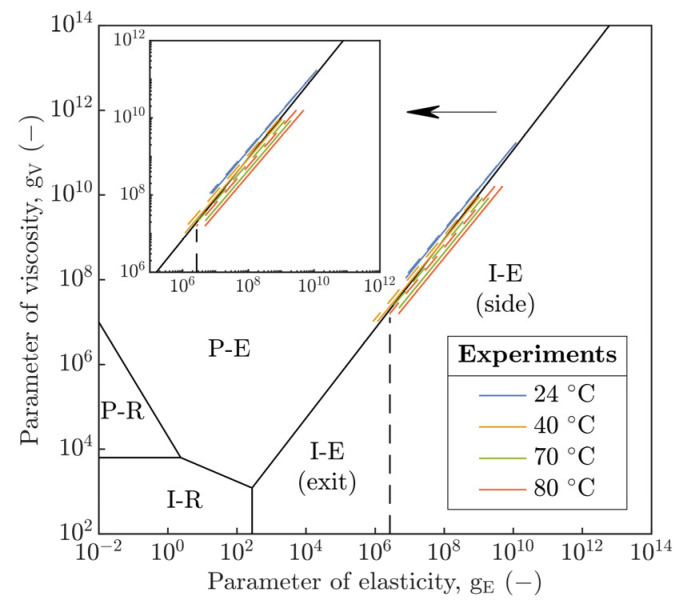
Hydrodynamic map of EHL modes for circular contact (k = 1).

Except for $\bar{U}$ and $\bar{W}$ parameters, the operating conditions of the current study were compared relative to the Johnson’s parameters [21] of viscosity g_V_ (Equation (SA1)) and elasticity g_E_ (Equation (SA2)) in the hydrodynamic map of EHL, see Figure 13. According to the hydrodynamic map, the contact was operated in the TR region [29,30] between the P-E and I-E modes with predisposition to the former or latter mode in the dependence on T that affects the η of the lubricant (see Table 2) as well as the E of polymer (see Table 6).

Based on Figure 13, it is evident that, at 24 °C, the circular contact was operated in the P-E mode characteristic for rigid contacts and usually relevant to the steel–steel, steel–glass or steel–sapphire contact pairs that exhibit a high contact pressure in the field of EHL. This could explain a good agreement of h_c_ as well as h_m_ compared to the hard H&D model [5] rather than to the soft one [19] at this temperature, see Figure 9a. At 40 °C, the contact was closest to the boundary of both EHL modes where Equation (SA4) or Equation (SA6) should not be directly applicable; however, a very good agreement of H_c_ with two soft EHL models [18,19] was recorded, see Figure 11. At 70 and 80 °C, the contact was shifted in the TR region closer to the I-E mode, which could be related to the increase in compliance of the PMMA polymer with increasing temperature.

The transition of h_m_ assumed by Hooke et al. [18] from the side lobes to the exit of the contact with increase in U_E_ was not attained, see Figure 13. Thus, h_m_ was always located at the side lobes of the horseshoe, see Figure 4. Compared to [31], the film thickness profiles, see Figure 5 and Figure 6, did not show a transition of the secondary minimum from the exit to the inlet of the contact due to the increase in U_E_ considering the viscoelastic response of the material. Then, the increase in U_E_ causes a rather elastic than viscoelastic response of the material, which is in accordance with Figure 2a,b. Thus, the stiffness of the compliant material increases.

However, a shrinkage in the longitudinal film thickness profiles was discovered with respect to the U_E_ increase, see Figure 7a,b. Here, the increase in U_E_ corresponds to a higher film thickness, where the shrinkage effect is more pronounced. This corresponds to the variations in ellipticity and the contact area for running contacts, while, for static contacts, it was mainly unchanged, see Table 5. Based on this, the question arises, how a distribution of the contact pressure in the compliant contact was affected. Nevertheless, the experimental approach dealing with the distribution of the contact pressure is somewhat challenging in the connection with the analysis of fluid-film thickness.

**Figure 14:**
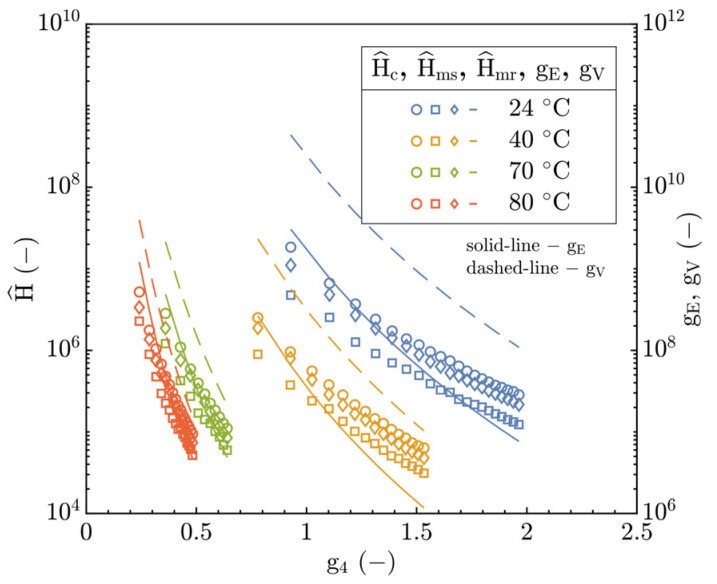
Parameters $\hat{H}$, g_E_ and g_V_ versus the Archard’s parameter of system g_4_.

The operating conditions of the experiments were compared not only for individual EHL modes but also for the expected TR region between the P-E and I-E modes in accordance with the research by Johnson [21], Jaffar [29] and Myers et al. [30]. Stated dimensionless parameters g_E_ (Equation (SA1)) and g_V_ (Equation (SA2)) were supplemented by the Archard’s parameter “of the system” g_4_ [22] which bounds the TR region to the interval where $g_{4}\langle \in \;0.1,\;2.2\rangle$, see Equation (SA3). In this connection, the film thickness prediction formulae were expressed using by g_E_, g_V_ parameters and the film thickness parameter ($\hat{H}=H\;{\left(\bar{W}/\bar{U}\right)}^{2}$). The central as well as minimum film thickness results expressed by ${\hat{H}}_{c}$, ${\hat{H}}_{mr}$ and ${\hat{H}}_{ms}$ parameters in Figure 14 are well consistent with the assumed interval for the TR region based on the g_4_ parameter in the interval from 0.2 to 1.95.

This could confirm the assumption of the operation of the contact in the TR region according to the hydrodynamic map of EHL, see Figure 13, and simultaneously, could justify the observed deviations of prediction models for the P-E and I-E modes of EHL applied to the TR region, see Figure 11. Relative to the g_E_ (right y-axis) in Figure 14, the film thickness development demonstrated a good quantitative agreement especially at T > 40 °C; however, at T ≤ 40 °C, it was significantly different. Contrary to the g_E_ parameter, the dependence of $\hat{H}$ on the g_V_ is represented by a significant disagreement for all inlet temperatures. This may again point to the dominant effect of constitutive material behavior.

For polymers, the constitutive material behavior corresponds to the viscoelastic state where the material response in dependence on time (or frequency) and the temperature of loading is manifested. Relative to tribology, these phenomena are expected especially for U_E_ ≠ 0, when the polymer (PMMA) is dynamically loaded. However, except for P-E EHL models, the I-E models similarly presume only the elastic response of the material, which is in contradiction with viscoelastic response of polymers.

From part A, elastic E′ as well as viscous E″ parts of the E*and tan (δ) were obtained from frequency and temperature sweep experiments based on nano-DMA and DMA methods. However, for the implementation to the EHL models, the question arises whether the E in these models can somehow be replaced by the E* obtained by both methods. The problem results from the fact that the E* corresponds to the frequency domain relative to the experimental methods employed. Thus, the conversion from the storage to the relaxation modulus (E_r_(t)), which characterizes viscoelastic properties in the time domain, is necessary.

**Figure 15:**
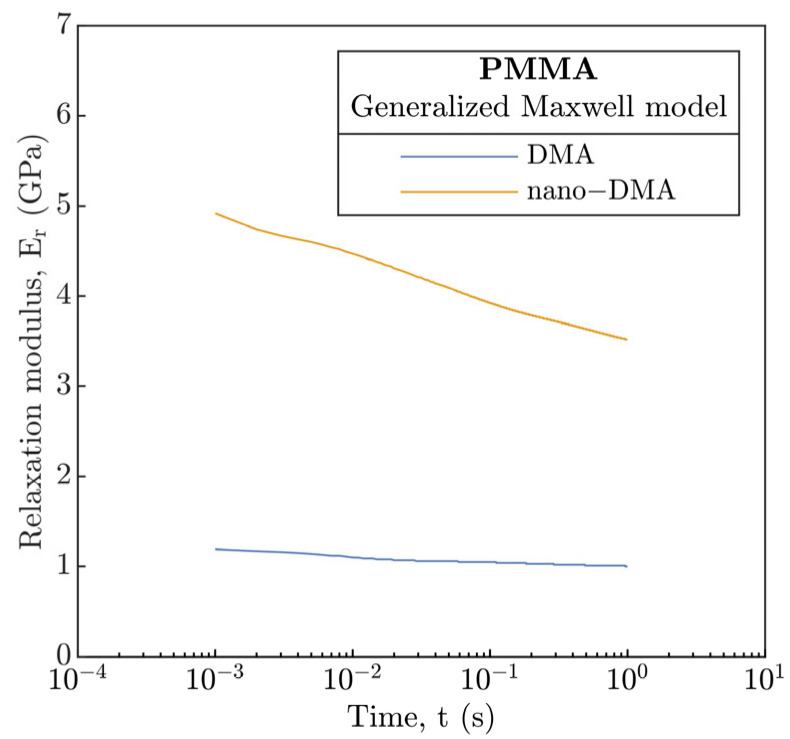
Relaxation modulus E_r_ based on generalized Maxwell model.

Hence, the generalized Maxwell model was used. The parameters of the seven-branch model are given in Tables SB1 and SB2, see Supplementary Information (SI)—B. The number of branches corresponds with significant peaks of E″ during the DMA temperature sweep experiments, see Figure 2b. In order to obtain model parameters (stiffnesses and relaxation times of the individual elements), the Prony series were fitted using the least squares method for the master-curves obtained from DMA and nano-DMA in Figure 2. Consequently, the E_r_ from nano-DMA was integrated into the EHL prediction models as a time-dependent function, substituting the conventional E. A time dependence (time needed to reach a given point on the disc corresponding to the distance equal to the R_c_) of the E_r_ is demonstrated in Figure 15.

The influence of E_r_ is evident from Figure 9 and Figure 10 where the H&D models for P-E and I-E mode of EHL were modified (H&D_m_, dashed-line). At 24 and 40 °C, the difference H_c_ from the modified soft and hard H&D_m_ models remains mainly unchanged relative to H&D, and H_c_ correlates best with the soft and hard H&D for the former and latter temperature, respectively. However, for 70 and 80 °C, the experimental H_c_ demonstrated a very good agreement with modified soft H&D_m_ model with only a slight deviation not exceeding 10% relative to soft H&D where a deviation over 60% was previously recorded. Interestingly, although the viscoelastic response characterized by E_r_ was included, the H_c_ also corresponds well with hard H&D and H&D_m_ models, especially at a temperature of 80 °C.

It is evident that the inlet temperature leads to a significant decrease in H_c_; however, the formation of film thickness in the TR region predominantly corresponds to the P-E mode rather than to the I-E mode. This is surprising relative to the constitutive material behavior of polymers, as well as the position of experiments close to the I-E mode, see Figure 13. However, this brings us back to the determination of the EHL mode, which may differ due to the integration of the E_r_ instead of the E.

The integration of the E_r_ in the hydrodynamic map, see Figure 13, shifts the operation conditions of the contact slightly toward the P-E mode of EHL in the TR region where the magnitude of shift with increasing temperature is enhanced. A similar shift would be seen in Figure 14 according to the Archard’s g_4_ parameter as well, but in a different manner. Here, the development of ${\hat{H}}_{c}$, ${\hat{H}}_{mr}$ and ${\hat{H}}_{ms}$ as well as g_E_ asymptotically approaches the boundary between the TR region and I-E mode of EHL and would converge to the value of 0.1 for g_4_, which is derived in [21] as the boundary point of TR region. However, the above points out to a significant discrepancy between the expected operation mode of EHL and I-E EHL models. Simultaneously, the described behavior may be a consequence of the viscoelastic response of the polymer, which causes a decrease in fluid-film thickness, not considered by the I-E EHL models.

In addition, several reasons can be found to explain the described deviations. Another possibility may be strain hardening at high contact pressures, which may not be evident in the DMA analysis. Conversely, the DMA analysis may have shown softening due to cyclic loading of one spot on the specimen. Hence, there are several reasons why the stiffness could be even lower. One of them could be a decrease by about 10% in the E measured by nano-DMA at the rolling path after the tribological experiment was performed in part B. Nevertheless, the softening effect should only be particularly noticeable at the temperature close to the T_g_ interval.

## 5. Conclusions

In the present study, the constitutive viscoelastic response of PMMA was experimentally determined relative to the tribology of compliant contacts operated in the EHL regime below the glass-transition temperature of the polymer. The viscoelastic response was evaluated by the generalized Maxwell model and implemented in I-E and P-E EHL models where only a purely elastic response of the contact solids has been assumed so far. Hence, a significant deviation often revealed for these models, especially for I-E when the temperature increases, then fluid-film thickness is overestimated. The operating mode of EHL was identified by hydrodynamic map (g_E_ and g_V_) and $\hat{H}$ and g_4_ parameters, and operating conditions of the formed circular contact were compared with research in the field of soft EHL. The main results can be summarized as follows:

The operating mode of the EHL has proved to be in the TR region close to the boundary between the P-E and I-E modes of the EHL according to the hydrodynamic map and the defined interval for the TR region via the Archard’s parameter g_4_. The shift of operating conditions to the vicinity of the I-E mode is manifested by an increase in the compliance of the circular contact after the inlet temperature is enhanced.

The implementation of a viscoelastic response of PMMA in the prediction models through the relaxation modulus reduced the deviation between the measured fluid-film thickness and the H&D model for the I-E and P-E modes. A decrease in deviation corresponds to an increase in contact compliance and the proximity of the operating conditions to the boundary between the TR region and the I-E mode.

The minimum film thickness was always localized at the side lobes of the horseshoe of the contact without the expected transition to the exit of the contact with increasing entrainment speed. The secondary minimum of film thickness at the inlet to the contact was not manifested as a consequence of the viscoelastic response of the polymer.

For the TR region, the pressure-viscosity effect of the lubricant should be included reflecting the mixed behavior at the boundary between the P-E and I-E modes of EHL, especially at lower temperatures. For T > 40 °C, the I-E EHL models generally predict the film thickness which is considerably overestimated even though the pressure-viscosity effect of the lubricant is always neglected.

The constitutive viscoelastic behavior should be considered in I-E EHL models; however, the variation in the material properties of individual polymers can make the generalization very difficult. Moreover, considering the lubricant rheology, this may be even more complex. The obtained results could be applicable to the design of polymer gears as well as to the optimalization of the lubrication management in the engineering applications where the rubbing surfaces of compliant and rigid solids interact under liquid lubrication conditions. For future research, the fluid-film thickness could be investigated in more detail considering the viscoelastic response of PMMA relative to the rheology of the lubricant.

## Figures and Tables

**Table 1:** Selected properties of specimens.

Specimen	Material	R_q_ (μm)	E (GPa)	υ (−)	T_g_ (°C)
Disc, plate and cylinder	PMMA	R_q1_ < 0.005	3.3	0.39	105–110
Ball	100Cr6	R_q2_ < 0.01	206	0.30	−

**Table 2:** Properties of reference lubricant—5P4E.

Lubricant—5P4E	T (40 °C)	T (100 °C)
Dynamic viscosity, η_0_ (Pa s)	0.490	0.015
Pressure–viscosity coefficient, α (GPa^−1^)	39.0	16.1

**Table 3:** Experimental conditions for nano-DMA and DMA measurements—part A.

Parameter	Nano-DMA	DMA
Specimen	plate	cylinder
DMA loading mode	indentation	compression
Dimensions of specimen	10 (w) × 10 (l) × 0.5 (t) mm	Ø 3 × 8 mm
Frequency, f	1–250 Hz	10^−3^–10^2^ Hz
Temperature, T	15, 20, 25, 30, 60, 80 °C	24, 40, 70, 80 °C

**Table 4:** Experimental conditions for fluid-film thickness measurements—part B.

Parameter	Value
Entrainment speed, U_E_	0.00025–0.8 m/s
Loading frequency, f_L_	~0.3–900 Hz
Normal load, W	35 N
Inlet temperature, T	24, 40, 70, 80 °C
Sliding-rolling ratio, SRR	0
Ellipticity of contact, *k*	1

**Table 5:** Dimensional parameters of static contacts at different inlet temperatures.

Inlet Temperature T, °C	24	40	70	80
Contact radius R_cx_, mm	0.439	0.466	0.503	0.528
Contact radius R_cy_, mm	0.440	0.458	0.501	0.527
Average R_c_, mm	0.439	0.462	0.502	0.527
Ellipticity *k*, −	0.997	1.015	1.002	1.001
Area A, mm^2^	0.606	0.671	0.788	0.874

**Table 6:** Reduced elastic modulus E_R_ and elastic modulus E_1_ of PMMA calculated for static contacts at different inlet temperatures.

Inlet Temperature T, °C	24	40	70	80
Reduced elastic modulus E_R_, GPa	7.853	6.746	5.301	4.538
Elastic modulus of PMMA E_1_, GPa	3.388	2.903	2.274	1.943

## Data Availability

The data concerned were obtained through the research of the author and reported exclusively in this article and Supplementary Information.

## Supplementary Materials

The following supporting information can be downloaded at: https://www.mdpi.com/article/10.3390/polym15112528/s1.

## Nomenclature

A	Area of contact: mm^2^
a_H_	Hertzian radius, μm
a_T_, b_T_	Horizontal and vertical shift factor (TTSP)
C_1_, C_2_	Constants for WLF equation, -, °C
E	Elastic modulus, MPa
E^*^	Complex modulus, MPa
E_A_	Viscoelastic activation energy, kJ mol^−1^
E′	Storage modulus, MPa
E″	Loss modulus, MPa
E_r_	Relaxation modulus, MPa
E_R_	Reduced elastic modulus, MPa
f	Frequency, Hz
f_L_	Frequency of loading, Hz
f_red_	Reduced frequency, Hz
g_4_	Archard’s parameter of the system
g_E_	Parameter of elasticity
g_V_	Parameter of viscosity
$\bar{G}$	Dimensionless material parameter
H	Dimensionless film thickness
$\hat{H}$	Film thickness parameter
H_c_	Dimensionless central film thickness
H_mr_	Dimensionless minimum film thickness at the exit of contact
H_ms_	Dimensionless minimum film thickness at the side lobes
h_c_	Central film thickness, nm
h_mr_	Minimum film thickness at the exit of contact, nm
h_ms_	Minimum film thickness at the side lobes, nm
i	Complex number
k	Ellipticity of contact (R_sx_/R_sy_)
p	Contact pressure, GPa
p_H_	Maximal Hertzian pressure, MPa
R	Gas constant, J mol^−1^ K^−1^
R’	Reduced curvature radius, mm
R_s_	Curvature radius of specimens, mm
R_c_	Radius of static and running contact, μm
R_q_	RMS roughness, μm
SRR	Sliding-rolling ratio
t	Time, s
T	Inlet temperature, °C
T_g_	Glass-transition temperature, °C
T_R_	Reference temperature, °C
tan δ	Damping/loss factor
U_E_	Entrainment speed, m/s
U	Surface speed of specimens, m/s
$\bar{U}$	Dimensionless speed parameter
W	Normal load, N
$\bar{W}$	Dimensionless load parameter
x, y	Coordinate perpendicular and parallel to U_E_
X, Y	Dimensionless x and y coordinate (x/a_H_ and y/a_H_)
α	Pressure-viscosity coefficient, GPa^−1^
δ	Phase angle between stress and strain, deg
η	Dynamic viscosity of lubricant, Pa s
η_0_	Dynamic viscosity of lubricant at atmospheric pressure, Pa s
ν	Poisson’s ratio of specimens
Subscripts	
1, 2	Index of PMMA disc and steel ball
alfa	Primary relaxation of polymer
beta, gamma, delta	Secondary relaxations of polymer
exp	Experimental values
mod	Model values

## References

[1] Gilbert M., Gilbert M. (2016). Plastics Materials: Introduction and Historical Development. Brydson’s Plastics Materials.

[2] Geyer R. (2020). A Brief History of Plastics. Mare Plasticum—The Plastic Sea.

[3] Dowson D. (1967). Paper 10: Elastohydrodynamics. Proceedings of the Institution of Mechanical Engineers, Conference Proceedings.

[4] Greenwood J.A. (1972). An extension of the Grubin theory of elastohydrodynamic lubrication. J. Phys. D. Appl. Phys..

[5] Hamrock B.J., Dowson D. (1977). Isothermal Elastohydrodynamic Lubrication of Point Contacts: Part III—Fully Flooded Results. J. Lubr. Technol..

[6] Dowson D. (1995). Elastohydrodynamic and micro-elastohydrodynamic lubrication. Wear.

[7] Höglund E. (1999). Influence of lubricant properties on elastohydrodynamic lubrication. Wear.

[8] Lugt P.M., Morales-Espejel G.E. (2011). A Review of Elasto-Hydrodynamic Lubrication Theory. Tribol. Trans..

[9] Meidav T. (1964). Viscoelastic Properties of the Standard Linear Solid. Geophys. Prospect..

[10] Chaudhri M. (2004). Impact breakage of semi-brittle spheres. Powder Technol..

[11] Harrass M., Friedrich K., Almajid A.A. (2010). Tribological behavior of selected engineering polymers under rolling contact. Tribol. Int..

[12] Friedrich K., Sue H.J., Liu P., Almajid A.A. (2011). Scratch resistance of high performance polymers. Tribol. Int..

[13] Ehret P., Dowson D., Taylor C.M. (1998). On lubricant transport conditions in elastohydrodynamic conjunctions. Proc. R. Soc. A Math. Phys. Eng. Sci..

[14] Krupka J., Dockal K., Krupka I., Hartl M. (2022). Elastohydrodynamic Lubrication of Compliant Circular Contacts near Glass-Transition Temperature. Lubricants.

[15] Krupka J., Dockal K., Krupka I., Hartl M. (2023). Polymer Lubrication: Pressure–Viscosity–Temperature Dependence of Film Thickness for Highly Loaded Compliant Contacts in Elastohydrodynamic Lubrication Regime. J. Tribol..

[16] Hartl M., Krupka I., Poliscuk R., Liska M., Molimard J., Querry M., Vergne P. (2001). Thin film colorimetric interferometry. Tribol. Trans..

[17] Mathiesen D., Vogtmann D., Dupaix R.B. (2014). Characterization and constitutive modeling of stress-relaxation behavior of Poly(methyl methacrylate) (PMMA) across the glass transition temperature. Mech. Mater..

[18] Hooke C.J. (1995). The Elastohydrodynamic Lubrication of Elliptical Point Contacts Operating in the Isoviscous Region. Proc. Inst. Mech. Eng. Part J J. Eng. Tribol..

[19] Hamrock B.J., Dowson D. (1978). Elastohydrodynamic Lubrication of Elliptical Contacts for Materials of Low Elastic Modulus I—Fully Flooded Conjunction. J. Lubr. Technol..

[20] Marx N., Guegan J., Spikes H.A. (2016). Elastohydrodynamic film thickness of soft EHL contacts using optical interferometry. Tribol. Int..

[21] Johnson K.L. (1970). Regimes of Elastohydrodynamic Lubrication. J. Mech. Eng. Sci..

[22] Esfahanian M., Hamrock B.J. (1991). Fluid-Film Lubrication Regimes Revisited. Tribol. Trans..

[23] Oliver W.C., Pharr G.M. (1992). An improved technique for determining hardness and elastic modulus using load and displacement sensing indentation experiments. J. Mater. Res..

[24] Mokhtari A., Tala-Ighil N., Masmoudi Y.A. (2022). Nanoindentation to Determine Young’s Modulus for Thermoplastic Polymers. J. Mater. Eng. Perform..

[25] De Deus J.F., Souza G.P., Corradini W.A., Atvars T.D.Z., Akcelrud L. (2004). Relaxations of Poly(methyl methacrylate) Probed by Covalently Attached Anthryl Groups. Macromolecules.

[26] De Vicente J., Stokes J.R., Spikes H.A. (2005). The Frictional Properties of Newtonian Fluids in Rolling–Sliding soft-EHL Contact. Tribol. Lett..

[27] Myant C., Spikes H.A., Stokes J.R. (2010). Influence of load and elastic properties on the rolling and sliding friction of lubricated compliant contacts. Tribol. Int..

[28] Fowell M.T., Myant C., Spikes H.A., Kadiric A. (2014). A study of lubricant film thickness in compliant contacts of elastomeric seal materials using a laser induced fluorescence technique. Tribol. Int..

[29] Jaffar M.J. (1989). Estimation of Minimum Thickness for Line Contacts in the Transition Region between the Isoviscous—Elastic and the Piezoviscous-Elastic Lubrication Regimes. Proc. Inst. Mech. Eng. Part C Mech. Eng. Sci..

[30] Myers T.G., Hall R.W., Savage M.D., Gaskell P.H. (1991). The Transition Region of Elastohydrodynamic Lubrication. Proc. R. Soc. London A Math. Phys. Eng. Sci..

[31] Putignano C., Dini D. (2017). Soft Matter Lubrication: Does Solid Viscoelasticity Matter?. ACS Appl. Mater. Interfaces.

