# Supplementary material for: Viscoelastic Response of Elastohydrodynamically Lubricated Compliant Contacts below Glass-Transition Temperature
Source: Polymers (Basel). 2023 May 30;15(11):2528. doi: 10.3390/polym15112528 (PMC10255053; doi:10.3390/polym15112528)
Supplement: Supplementary file 1 [file polymers-15-02528-s001.zip › polymers-2348671-supplementary.pdf]

Supplementary Information for

Viscoelastic response of elastohydrodynamically lubricated compliant contacts below glass-transition temperature

Jiri Krupka [1,*], Krystof Dockal[2], Tomas Sedlacek[3], David Rebenda[1,4], Ivan Krupka[1] and Martin Hartl[1]

[1] Faculty of Mechanical Engineering, Brno University of Technology, Technicka 2896/2, 616 69 Brno, Czech Republic; jiri.krupka@vut.cz; (J.K.); david.rebenda@vut.cz (D.R.); krupka@fme.vutbr.cz (I.K.); hartl@fme.vutbr.cz (M.H.)
[2] HVM Plasma spol. s.r.o., Na Hutmance 2, 158 00 Praha 5, Czech Republic; krystof.dockal@hvm.cz (K.D.)
[3] Centre of Polymer Systems, Tomas Bata University in Zlin, Trida Tomase Bati 5678, 760 01 Zlin, Czech Republic; sedlacek@utb.cz; (T.S.)
[4] Footwear Research Centre, University Institute, Tomas Bata University in Zlin, Nad Ovcirnou IV 3685, 760 01 Zlin, Czech Republic; rebenda@utb.cz (D.R.)
* Correspondence: jiri.krupka@vut.cz; (J.K.)

A–Background

EHL was a subject of intense research for decades when the hard and soft modes of EHL were distinguished as Piezoviscous-elastic (P-E) [5] and Piezoviscous-rigid (P-R), and Isoviscous-elastic (I-E) [32] and Isoviscous rigid (I-R), respectively. For the definition of individual EHL modes, Johnson [21], Hamrock and Dowson [19], Esfahanian and Hamrock [22], Hooke et al. [18,33] and others [29–30] derived hydrodynamic maps of EHL modes, which put in the contrast the operating conditions relative to viscosity g_V and elasticity g_E parameters, see Eq. (SA1–SA2), for different contact geometries—line, circular (k = 1) and elliptical (k < 1 or k > 1) contacts. Both parameters were supplemented by the Archard's parameter of the system g_4, see Eq. (SA3), which additionally defines the transition region (TR) between P-E and I-E modes to the interval $g_4 \in \langle 0.1, 2.2 \rangle$.

$$g_V = \frac{\overline{G}\,\overline{W}^3}{\overline{U}^2} \tag{SA1}$$

$$g_E = \frac{\overline{W}^{8/3}}{\overline{U}^2} \tag{SA2}$$

$$g_4 = g_V\, {g_E}^{-1.5} \tag{SA3}$$

where $\overline{U} = \frac{U\,\eta_0}{E_R\,R'}$; $\overline{W} = \frac{W}{E_R R'^2}$, $\overline{G} = E_R\alpha$. All symbols are stated in [19,21] and in the Nomenclature of this paper.

Briefly, the classical EHL theory [3–8] is characterized by three crucial mechanisms influencing the formation of the central (h_c) and minimum (h_m) film thickness in the contact. The first two mechanisms cover the pressure–viscosity effect [34–35] and the hydrodynamic effect of the fluid according to the Reynolds equation [13,36] and thus represent the rheological response of the lubricant to the operating conditions. These mechanisms usually have a positive impact on the increase in fluid-film thickness in the EHL regime.

The third mechanism is represented by the constitutive material response of the solids, which commonly results in the elastic deformation of the rubbing surfaces after the non-conformal contact is loaded [37–38]. This is typical for the P-E mode of EHL where the elastic deformation of high elastic modulus (E) solids, such as metals, ceramics or glass, leads to high contact pressures (p) up to several gigapascals (p ≈ 3–4 GPa) [39–40]. Hence, the high contact pressure has a significant effect on the pressure–viscosity relationship, and thus on the increase in film thickness in dependence on the used lubricant characterized by the pressure–viscosity coefficient α [40–41]. The rigid modes of EHL are beyond the focus of this paper due to the low relevance of the third mechanism.

Contrary to the P-E mode, the I-E mode of EHL is distinguished by low E of contact solids, such as elastomers and polymers, and the contact is rather compliant [32,42]. Moreover, the low contact pressure (usually p < 1 GPa) is insufficient to change the dynamic viscosity (η) in this mode. Moreover, the low stiffness of compliant solids, attributed to E, contributes to the significant increase in width of contact strip for the line contacts [43] and the contact radius (R_c) for circular [20,27,44] / elliptical [18,28] contacts when the contact is loaded.

In practical applications, such as transmissions and mechanisms, the contact of the meshing gears made of different materials is frequently provided, where E of the steel pinion is significantly higher than for the wheel gear, usually made from the engineering [11] and high-performance [12] polymers. Thus, the contact pair represents the rigid (hard) and compliant (soft) solids. With respect to this, the operation of the contact in the TR region [22,30] between the P-E and I-E modes of the EHL regime is expected; however, only limited research in this field of EHL has been performed so far. Moreover, the validity of the prediction models [5,18–20] of the film thickness corresponding to the P-E and I-E modes of EHL for the TR region has not been sufficiently validated yet.

Moreover, the viscoelastic response of a compliant material to the formation of film thickness [45–46] has been often neglected. The prediction formulae for dimensionless H_c as well as H_m film thickness in circular contact related to the P-E and I-E modes for H&D model [5,19] are described by Eqs. (SA4) and (SA5) and Eqs. (SA6) and (SA7), respectively. Alternatively, these prediction formulae could be expressed using the g_V (Eq. (SA1)) and g_E (Eq. (SA2)) parameters.

P-E mode:

$$H_c = \frac{h_c}{R'} = 1.9\,\bar{U}^{0.67}\,\bar{W}^{-0.067}\,\bar{G}^{0.53} = 2.55 g_V{}^{0.53} g_E{}^{0.13}\left(\frac{\bar{U}}{\bar{W}}\right)^2 \tag{SA4}$$

$$H_m = \frac{h_m}{R'} = 1.8\,\bar{U}^{0.68}\,\bar{W}^{-0.073}\,\bar{G}^{0.49} = 1.69 g_V{}^{0.49} g_E{}^{0.17}\left(\frac{\bar{U}}{\bar{W}}\right)^2 \tag{SA5}$$

I-E mode:

$$H_c = \frac{h_c}{R'} = 3.3\,\bar{U}^{0.64}\,\bar{W}^{-0.22} = 5.08\, g_E{}^{0.67}\left(\frac{\bar{U}}{\bar{W}}\right)^2 \tag{SA6}$$

$$H_m = \frac{h_m}{R'} = 2.8\,\bar{U}^{0.65}\,\bar{W}^{-0.21} = 3.28\, g_E{}^{0.67}\left(\frac{\bar{U}}{\bar{W}}\right)^2 \tag{SA7}$$

A phenomenon of viscoelastic behavior is achieved after the material exhibits a nonlinear (non-Hookean) response in time in relation to the given constant stress or strain corresponding to the stress–strain dependence [47–48]. The viscoelastic response of the material is frequently numerically simulated by standard linear solid models (SLS) such as Maxwell's, Zener's and Kelvin–Voight's models, where dampers and springs in serial or parallel arrangement invoke a time-varying response [9,49]. Thus, the original elastic modulus is substituted by the relaxation or complex modulus with regard to the temperature or time (or frequency) domain, respectively. The complex modulus E^* includes two main components: the elastic part, the storage modulus (E′) as well as the viscous part, the loss modulus (E′′), see Eq. (SA8).

$$E^* = E' + i\,E'' \tag{SA8}$$

$$\tan\delta = \frac{\sin\delta}{\cos\delta} = \frac{E''}{E'} \tag{SA9}$$

The ratio between them represents the damping / loss factor tan δ, see Eq. (SA9), corresponding to the stored energy in the material and the loss energy converted (energy

dissipated due to internal friction of molecules in the polymer structure) to the heat after the polymer is tempered from glassy state ($T << T_g$) and viscoelastic state ($T \leq T_g$.) to the rubbery plateau where $T > T_g$. The individual states define a macromolecular arrangement, and the reciprocal motion of molecules of the polymer chains (bonds) relative to the internal polymer structure and operating conditions [50]. According to the internal structure, the amorphous (PMMA, PC), semi-crystalline (PA66, POM, PEEK) and crystalline polymers demonstrate a significantly different behavior [51–52] in individual states with the rising temperature where the amorphous polymers prove a substantial temperature reliance rather than semi-crystalline and crystalline ones.

Furthermore, the constitutive temperature dependent behavior of polymers is characterized by the relaxation transitions labeled alfa, beta, gamma and delta in a decreasing order to the temperature where the main-chain and individual side-chains of polymer internal structure are rearranged, and the material demonstrates a different viscoelastic response [25]. The primary alfa-relaxation relates to the glass-transition temperature T_g of polymer in the viscoelastic state when the motion of molecules of the main-chain (chain backbone) is enhanced [17,53]. However, beta, gamma and delta—secondary relaxations—take place in the interval below T_g in the glassy state and they especially result from the side-chains molecular motions of the polymer [54] as well as heteroatom motions in the main-chain [55]. Simultaneously, the mobility of the main-chain (chain backbone) molecules decreases relative to the alfa-relaxation state but does not fully vanish [56]. The internal energy for initialization of the primary or secondary relaxation transitions of molecules corresponds to the viscoelastic activation energy (E_A) according to the inverse alphabetical order, i.e., $E_{A,\,beta} < E_{A,\,alfa}$ [57].

In practice, the E′ and E′′ moduli are often experimentally determined by the creep and relaxation tests using the nanoindentation [9,58] or by a dynamic mechanical analysis (DMA) [31,59] where the δ represents the phase angle between stress and strain corresponding to the interval of $\delta \in \langle 0, \pi/2 \rangle$. With increasing value of tan δ (f, T), the polymer exhibits more compliance (inverted parameter to the stiffness) in the mechanical cyclic loading, and thus, the material capability to damp the impacts and vibrations is maximal [60].

The influence of the time (or frequency f) of loading and temperature *T* can be combined to obtain the complex material response of the polymers by the time-temperature superposition principle (TTSP) [61,62]. With respect to this, the Arrhenius (Eq. (SA10)) and the William–Landel–Ferry (WLF) (Eq. (SA11)) equations are predominantly used in the temperature interval $T < T_g$ and $T \geq T_g$, respectively, to determine the time–temperature transformation by horizontal (a_T) and vertical (b_T) shift factors [54,57]. This is associated to the selected reference temperature T_R. However, the b_T is often omitted in TTSP.

$$\ln a_T = \frac{E_A}{R}\left(\frac{1}{T} - \frac{1}{T_R}\right) \tag{SA10}$$

$$\log a_T = -\frac{C_1\,(T - T_R)}{C_2 + (T - T_R)} \tag{SA11}$$

where C_1, C_2 are constants, for WLF, appropriate to the individual polymers, and R (8.164 J mol^{-1} K^{-1}) is a universal gas constant.

When the influence of time (or frequency) and temperature on the E^* is reciprocally equivalent, the polymer is considered as thermorheologically simple covering a single relaxation time spectra H(τ) [61] where τ is the relaxation time. Hence, the viscoelastic deformation is governed only by a single molecular process. Consequently, the sole master-curve (MC) of the E^* or tan δ covering the wide temperature and time (or frequency) interval can be derived for the individual thermorheologically simple polymers by a_T. This provides necessary input data to include the viscoelastic response of the material into the prediction models of fluid-film thickness, especially in the I-E mode of EHL. Otherwise, the polymer is thermorheologically complex [63], and the TTSP is not applicable [56].

B—Generalized Maxwell model (Prony series)

Equilibrium relaxation modulus—E_∞

Table SB1. Parameters of the generalized Maxwell model – nDMA (E_∞ = 3.43 GPa)

Modes j=1…n	Linear spring modulus, E_j (MPa)	Relaxation time, τ_j (s)
1	8.27E+02	9.10E-07
2	3.80E+02	2.58E-05
3	1.57E+02	2.14E-04
4	7.10E+02	7.35E-04
5	3.37E+02	1.03E-02
6	5.24E+02	5.53E-02
7	4.81E+02	5.71E-01

Table SB2. Parameters of the generalized Maxwell model—DMA (E_∞ = 0.81 GPa)

Modes j=1…n	Linear spring modulus, E_j (MPa)	Relaxation time, τ_j (s)
1	4.98E+01	1.84E-01
2	1.35E+02	7.70E-03
3	1.90E+01	3.09E+00
4	2.97E+01	3.10E+00
5	2.90E+01	1.44E+01
6	1.28E-05	9.45E+02
7	1.23E+02	1.00E+03

References

3. Dowson, D. Paper 10: Elastohydrodynamics. Proc. Inst. Mech. Eng. Conf. Proc. 1967, 182, 151–167, doi:10.1243/PIME_CONF_1967_182_014_02.
4. Greenwood, J.A. An extension of the Grubin theory of elastohydrodynamic lubrication. J. Phys. D. Appl. Phys. 1972, 5, 309, doi:10.1088/0022-3727/5/12/309.
5. Hamrock, B.J.; Dowson, D. Isothermal Elastohydrodynamic Lubrication of Point Contacts: Part III—Fully Flooded Results. J. Lubr. Technol. 1977, 99, 264–275.
6. Dowson, D. Elastohydrodynamic and micro-elastohydrodynamic lubrication. Wear 1995, 190, 125–138, doi:10.1016/0043-1648(95)06660-8.
7. Höglund, E. Influence of lubricant properties on elastohydrodynamic lubrication. Wear 1999, 232, 176–184, doi:10.1016/S0043-1648(99)00143-X.
8. Lugt, P.M.; Morales-Espejel, G.E. A Review of Elasto-Hydrodynamic Lubrication Theory. Tribol. Trans. 2011, 54, 470–496, doi:10.1080/10402004.2010.551804.
9. MEIDAV, T. VISCOELASTIC PROPERTIES OF THE STANDARD LINEAR SOLID*. Geophys. Prospect. 1964, 12, 80–99, doi:10.1111/j.1365-2478.1964.tb01891.x.
11. Harrass, M.; Friedrich, K.; Almajid, A.A. Tribological behavior of selected engineering polymers under rolling contact. Tribol. Int. 2010, 43, 635–646, doi:10.1016/j.triboint.2009.10.003.
12. Friedrich, K.; Sue, H.J.; Liu, P.; Almajid, A.A. Scratch resistance of high performance polymers. Tribol. Int. 2011, 44, 1032–1046, doi:10.1016/j.triboint.2011.04.008.
13. Ehret, P.; Dowson, D.; Taylor, C.M. On lubricant transport conditions in elastohydrodynamic conjunctions. Proc. R. Soc. A Math. Phys. Eng. Sci. 1998, 454, 763–787, doi:10.1098/rspa.1998.0185.
17. Mathiesen, D.; Vogtmann, D.; Dupaix, R.B. Characterization and constitutive modeling of stress–relaxation behavior of Poly(methyl methacrylate) (PMMA) across the glass transition temperature. Mech. Mater. 2014, 71, 74–84, doi:10.1016/j.mechmat.2014.01.003.
18. Hooke, C.J. The Elastohydrodynamic Lubrication of Elliptical Point Contacts Operating in the Isoviscous Region. Proc. Inst. Mech. Eng. Part J J. Eng. Tribol. 1995, 209, 225–234, doi:10.1243/PIME_PROC_1995_209_433_02.
19. Hamrock, B.J.; Dowson, D. Elastohydrodynamic Lubrication of Elliptical Contacts for Materials of Low Elastic Modulus I—Fully Flooded Conjunction. J. Lubr. Technol. 1978, 100, 236–245, doi:10.1115/1.3453152.

20. Marx, N.; Guegan, J.; Spikes, H.A. Elastohydrodynamic film thickness of soft EHL contacts using optical interferometry. Tribol. Int. 2016, 99, 267–277, doi:10.1016/j.triboint.2016.03.020.
21. Johnson, K.L. Regimes of Elastohydrodynamic Lubrication. J. Mech. Eng. Sci. 1970, 12, 9–16, doi:10.1243/JMES_JOUR_1970_012_004_02.
22. Esfahanian, M.; Hamrock, B.J. Fluid-Film Lubrication Regimes Revisited. Tribol. Trans. 1991, 34, 628–632, doi:10.1080/10402009108982081.
25. de Deus, J.F.; Souza, G.P.; Corradini, W.A.; Atvars, T.D.Z.; Akcelrud, L. Relaxations of Poly(methyl methacrylate) Probed by Covalently Attached Anthryl Groups. Macromolecules 2004, 37, 6938–6944, doi:10.1021/ma049941c.
27. Myant, C.; Spikes, H.A.; Stokes, J.R. Influence of load and elastic properties on the rolling and sliding friction of lubricated compliant contacts. Tribol. Int. 2010, 43, 55–63, doi:10.1016/j.triboint.2009.04.034.
28. Fowell, M.T.; Myant, C.; Spikes, H.A.; Kadiric, A. A study of lubricant film thickness in compliant contacts of elastomeric seal materials using a laser induced fluorescence technique. Tribol. Int. 2014, 80, 76–89, doi:10.1016/j.triboint.2014.05.028.
29. Jaffar, M.J. Estimation of Minimum Thickness for Line Contacts in the Transition Region between the Isoviscous—Elastic and the Piezoviscous-Elastic Lubrication Regimes. Proc. Inst. Mech. Eng. Part C Mech. Eng. Sci. 1989, 203, 379–386, doi:10.1243/PIME_PROC_1989_203_131_02.
30. Myers, T.G.; Hall, R.W.; Savage, M.D.; Gaskell, P.H. The Transition Region of Elastohydrodynamic Lubrication. Proc. R. Soc. London A Math. Phys. Eng. Sci. 1991, 432, 467–479, doi:10.1098/rspa.1991.0026.
31. Putignano, C.; Dini, D. Soft Matter Lubrication: Does Solid Viscoelasticity Matter? ACS Appl. Mater. Interfaces 2017, 9, 42287–42295, doi:10.1021/acsami.7b09381.
32. Hooke, C.J.; O'Donoghue, J.P. Elastohydrodynamic Lubrication of Soft, Highly Deformed Contacts. J. Mech. Eng. Sci. 1972, 14, 34–48, doi:10.1243/JMES_JOUR_1972_014_008_02.
33. Hooke, C.J. The Elastohydrodynamic Lubrication of Heavily Loaded Contacts. J. Mech. Eng. Sci. 1977, 19, 149–156, doi:10.1243/JMES_JOUR_1977_019_034_02.
34. Vergne, P.; Berthe, D. Paper X(i) Pressure viscosity and compressibility of different mineral oils. In Tribology Series; 1987; Vol. 11, pp. 309–315.
35. Bair, S. Pressure–viscosity response in the inlet zone for quantitative elastohydrodynamics. Tribol. Int. 2016, 97, 272–277, doi:10.1016/j.triboint.2016.01.037.
36. Ponjavic, A.; Chennaoui, M.; Wong, J.S.S. Through-thickness velocity profile measurements in an elastohydrodynamic contact. Tribol. Lett. 2013, 50, 261–277, doi:10.1007/s11249-013-0118-x.
37. Dowson, D.; Hamrock, B.J. Numerical Evaluation of the Surface Deformation of Elastic Solids Subjected to a Hertzian Contact Stress. A S L E Trans. 1976, 19, 279–286, doi:10.1080/05698197608982804.
38. Snoeijer, J.H.; Eggers, J.; Venner, C.H. Similarity theory of lubricated Hertzian contacts. Phys. Fluids 2013, 25, 1–7, doi:10.1063/1.4826981.
39. Krupka, I.; Kumar, P.; Bair, S.; Khonsari, M.M.; Hartl, M. The Effect of Load (Pressure) for Quantitative EHL Film Thickness. Tribol. Lett. 2010, 37, 613–622, doi:10.1007/s11249-009-9559-7.
40. Roelands, C.J.A.; Vlugter, J.C.; Waterman, H.I. The viscosity-temperature-pressure relationship of lubricating oils and its correlation with chemical constitution. J. Fluids Eng. Trans. ASME 1963, 85, 601–607, doi:10.1115/1.3656919.
41. Bair, S.; Kottke, P. Pressure-viscosity relationships for elastohydrodynamics. Tribol. Trans. 2003, 46, 289–295, doi:10.1080/10402000308982628.
42. Biswas, S.; Snidle, R.W. Elastohydrodynamic Lubrication of Spherical Surfaces of Low Elastic Modulus. J. Lubr. Technol. 1976, 98, 524–529, doi:10.1115/1.3452912.
43. Hooke, C.J.; Huang, P. Elastohydrodynamic lubrication of soft viscoelastic materials in line contact. Proc. Inst. Mech. Eng. Part J J. Eng. Tribol. 1997, 211, 185–194, doi:10.1243/1350650971542417.
44. Gasni, D.; Wan Ibrahim, M.K.; Dwyer-Joyce, R.S. Measurements of lubricant film thickness in the iso-viscous elastohydrodynamic regime. Tribol. Int. 2011, 44, 933–944, doi:10.1016/j.triboint.2011.03.010.
45. Essink, M.H.; Pandey, A.; Karpitschka, S.; Venner, C.H.; Snoeijer, J.H. Regimes of soft lubrication. J. Fluid Mech. 2021, 915, A49, doi: 10.1017/jfm.2021.96.
46. Zhao, Y.; Liu, H.C.; Morales-Espejel, G.E.; Venner, C.H. Effects of solid viscoelasticity on elastohydrodynamic lubrication of point contacts. Tribol. Int. 2022, 171, 107562, doi:10.1016/j.triboint.2022.107562.
47. Chen, D.T.N.; Wen, Q.; Janmey, P.A.; Crocker, J.C.; Yodh, A.G. Rheology of Soft Materials. Annu. Rev. Condens. Matter Phys. 2010, 1, 301–322, doi:10.1146/annurev-conmatphys-070909-104120.
48. Wineman, A. Nonlinear Viscoelastic Solids—A Review. Math. Mech. Solids 2009, 14, 300–366, doi:10.1177/1081286509103660.
49. Christöfl, P.; Czibula, C.; Berer, M.; Oreski, G.; Teichert, C.; Pinter, G. Comprehensive investigation of the viscoelastic properties of PMMA by nanoindentation. Polym. Test. 2021, 93, 106978, doi:10.1016/j.polymertesting.2020.106978.
50. Fetters, L.J.; Lohse, D.J.; Richter, D.; Witten, T.A.; Zirkel, A. Connection between Polymer Molecular Weight, Density, Chain Dimensions, and Melt Viscoelastic Properties. Macromolecules 1994, 27, 4639–4647, doi:10.1021/ma00095a001.
51. Dos Santos, W.N.; De Sousa, J.A.; Gregorio, R. Thermal conductivity behaviour of polymers around glass transition and crystalline melting temperatures. Polym. Test. 2013, 32, 987–994, doi:10.1016/j.polymertesting.2013.05.007.
52. Djukic, S.; Bocahut, A.; Bikard, J.; Long, D.R. Mechanical properties of amorphous and semi-crystalline semi-aromatic polyamides. Heliyon 2020, 6, doi:10.1016/j.heliyon.2020.e03857.

53. Sane, S.B.; Knauss, W.G. The time-dependent bulk response of Poly (Methyl Methacrylate). Mech. Time-Dependent Mater. 2001, 5, 293–324, doi:10.1023/A:1012517421899.
54. Higgenbotham-bertolucci, P.R.; Gao, H.; Harmon, J.P. Creep and stress relaxation in methacrylate polymers: Two mechanisms of relaxation behavior across the glass transition region. Polym. Eng. Sci. 2001, 41, 873–880, doi:10.1002/pen.10785.
55. Wu, S. Secondary relaxation, brittle–ductile transition temperature, and chain structure. J. Appl. Polym. Sci. 1992, 46, 619–624, doi:10.1002/app.1992.070460408.
56. Klompen, E.T.J.; Govaert, L.E. Nonlinear viscoelastic behaviour of thermorheologically complex materials: A modelling approach. Mech. Time-Dependent Mater. 1999, 3, 49–69, doi:10.1023/A:1009853024441.
57. Federico, C.E.; Bouvard, J.L.; Combeaud, C.; Billon, N. Large strain/time dependent mechanical behaviour of PMMAs of different chain architectures. Application of time-temperature superposition principle. Polymer (Guildf). 2018, 139, 177–187, doi:10.1016/j.polymer.2018.02.021.
58. Mazeran, P.E.; Beyaoui, M.; Bigerelle, M.; Guigon, M. Determination of mechanical properties by nanoindentation in the case of viscous materials. Int. J. Mater. Res. 2012, 103, 715–722, doi:10.3139/146.110687.
59. Menard, K.P.; Menard, N. Dynamic Mechanical Analysis DYNAMICAL MECHANICAL ANALYSIS. Encycl. Anal. Chem. 2017, 1–22, doi:10.1002/9780470027318.a2007.pub3.
60. Schiessel, H.; Metzler, R.; Blumen, A.; Nonnenmacher, T.F. Generalized viscoelastic models: Their fractional equations with solutions. J. Phys. A Gen. Phys. 1995, 28, 6567–6584, doi:10.1088/0305-4470/28/23/012.
61. Colby, R.H. Breakdown of time-temperature superposition in miscible polymer blends. Polymer (Guildf). 1989, 30, 1275–1278, doi:10.1016/0032-3861(89)90048-7.
62. Barbero, E.J. Time-temperature-age superposition principle for predicting long-term response of linear viscoelastic materials. Creep Fatigue Polym. Matrix Compos. 2010, 48–69, doi:10.1533/9780857090430.1.48.
63. Brinson, L.C.; Knauss, W.G. Thermorheologically complex behavior of multi-phase viscoelastic materials. J. Mech. Phys. Solids 1991, 39, 859–880, doi:10.1016/0022-5096(91)90009-D.
